# Supplementary figures and images for: A neural network‐based 2D/3D image registration quality evaluator for pediatric patient setup in external beam radiotherapy
Source: J Appl Clin Med Phys. 2016 Jan 8;17(1):22–33. doi: 10.1120/jacmp.v17i1.5235 (PMC5690212; doi:10.1120/jacmp.v17i1.5235)

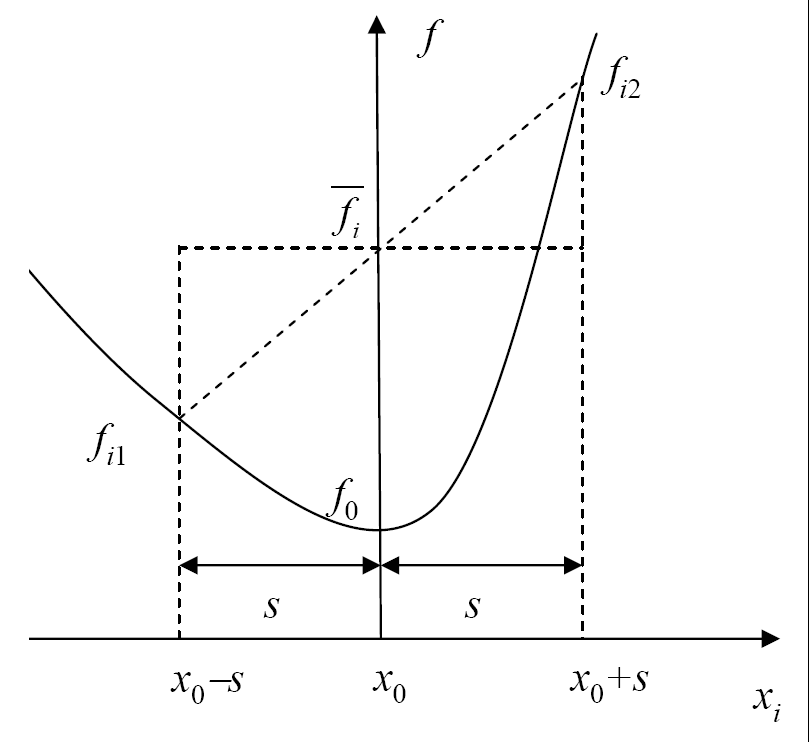

Supplement: Supplementary file 1 — Supplementary Material [file ACM2-17-022-s001.png]
