# Supplementary material for: A neural network‐based 2D/3D image registration quality evaluator for pediatric patient setup in external beam radiotherapy
Source: J Appl Clin Med Phys. 2016 Jan 8;17(1):22–33. doi: 10.1120/jacmp.v17i1.5235 (PMC5690212; doi:10.1120/jacmp.v17i1.5235)
Supplement: Supplementary file 2 — Supplementary Material [file ACM2-17-022-s002.docx]

**ABSTRACT**

**Purpose:** To develop a neural network based registration quality evaluator (RQE) that can improve the 2D/3D image registration robustness for pediatric patient setup in external beam radiotherapy.

**Methods and Materials:** Orthogonal x-ray daily setup images of six pediatric patients with brain tumors receiving proton therapy treatments were retrospectively registered with their treatment planning computed tomography (CT) images. A neural network based pattern classifier was used to determine whether a registration solution is successful based on geometric features of the similarity measure values near the point-of-solution. Supervised training and test datasets were generated by rigidly registering a pair of orthogonal daily setup x-ray images to the treatment planning CT. The best solution for each registration task was selected from 50 optimizing attempts that differed only by the randomly generated initial transformation parameters. The distance from each individual solution to the best solution in the normalized parametrical space was compared to a user-defined error tolerance to determine whether that solution was acceptable. The supervised training was then used to train the RQE. Performance of the RQE was evaluated using test dataset consisting of registration results that were not used in training. The RQE was integrated with our in-house 2D/3D registration system and its performance was evaluated using the same patient dataset.

**Results:** With an optimized sampling step size, i.e., 5 mm, in the feature space, the RQE has the sensitivity and the specificity in the ranges of 0.865-0.964 and 0.797-0.990, respectively, when used to detect registration error with mean voxel displacement (MVD) greater than 1 mm. The trial-to-acceptance ratio of the integrated 2D/3D registration system, for all patients, is equal to 1.48. The final acceptance ratio is 92.4%.

**Conclusions:** The proposed RQE can potentially be used in a 2D/3D rigid image registration system to improve the overall robustness by rejecting unsuccessful registration solutions. The RQE is not patient specific, so a single RQE can be constructed and used for a particular application (e.g., the registration for images acquired on the same anatomical site). Implementation of the RQE in a 2D/3D registration system is clinically feasible.

**Keywords:** image registration, neural network, image guided radiation therapy, brain tumor, registration quality assurance

**PACS numbers:** 87.57.nj, 87.85.dq, 87.55.Qr

I. **INTRODUCTION**

In external beam radiation therapy, registration of 2D x-ray projection images and 3D CT images has been used clinically for patient setup and motion mitigation.[^1^](#_ENREF_1), [^2^](#_ENREF_2) Compared to 3D/3D registration-based patient setup strategies, the 2D/3D registration method does not require lengthy 3D image acquisition on the daily basis and, more importantly, reduces imaging dose to the patient. The stochastic risk of carcinogenesis caused by imaging dose is a major concern for pediatric patients because of their longer life expectancy. For patient setup, although a number of dose reduction strategies for the CBCT imaging have been proposed[^3^](#_ENREF_3), a 2D x-ray imaging technique may still be a better choice if the 2D registration yields solutions that are clinically acceptable. At the present time, CBCT imaging system is not available in proton therapy systems. Patient setup for proton therapy patients relies on an x-ray imaging system with orthogonal projection geometry and a 2D image matching technique. Development of a reliable automated 2D/3D registration system may potentially reduce the patient setup time and improve the setup accuracy.

2D/3D registration approaches for patient positioning have been investigated[^4-8^](#_ENREF_4) and were also implemented in commercial systems such as the BrainLAB ExacTrac x-ray imaging system (BrainLAB, Feldkirchen, Germany), the Accuray CyberKnife Xsight system (Accuray, Sunnyvale, CA), and the MEDCOM VeriSuite system (Darmstadt, Germany) for proton therapy systems. These systems align two x-ray (orthogonal or oblique) projection images to a treatment planning CT image dataset and compute six transformation (3 translational and 3 rotational) parameters. When used with a robotic patient couch that is able to move in 5 or 6-degree of freedom, the system can achieve better patient setup correction compared to traditional correction strategy that are based only on translations.

2D/3D registration requires computation of digitally reconstructed radiographs (DRR) from a 3D CT dataset. A few years ago the computation burden of this step was a major obstacle that prevented the clinical implementation of such systems. However, this problem has been overcome by advances of graphics card-based computation techniques.[^7^](#_ENREF_7), [^9^](#_ENREF_9) The calculation time required for an intensity-based 2D/3D registration reduced from a few hours using CPUs[^4^](#_ENREF_4) to several seconds using graphics processing units (GPUs). With the technology of graphics-based computation continuously evolving, the speed of registration is no longer a concern. System reliability in terms of registration accuracy and robustness for a variety of anatomical disease sites has become the current challenge for a clinic 2D/3D registration system.

An intensity-based 2D/3D registration algorithm aligns the acquired planar images with the computed DRRs by minimizing a cost function. An optimization process can be trapped in a local minimum due to the complexity of patient anatomy, image noise and artifacts, organ deformation, and the lack of monotonicity of the similarity function, which results in incorrect solutions. The frequency of such failures can depend on the initial conditions of 2D/3D registration. Unsuccessful registration rate has been reported to be as high as 32% when initial shifts were between 6 to 16 mm.[^10^](#_ENREF_10) Traditionally, evaluation of the acceptability of a registration solution is carried out by visually comparing the overlapped source and target images. This method is subjective and lacks consistency. Pattern classifier-based registration quality evaluation methods have been proposed by Wu and colleagues to identify local minima and avoid premature termination, and have been successfully applied to 2D/2D[^11^](#_ENREF_11) and 3D/3D[^12^](#_ENREF_12) registration applications. Based on phantom and patient tests, they have demonstrated that the registration quality evaluator (RQE) could reject registration results with errors larger than a user-defined tolerance.

In this paper, we extended the use of RQE to 2D/3D registration applications and developed a neural network based RQE to handle the multi-dimensional feature vectors of the training dataset. Compared to the 3D/3D RQE, the construction of a 2D/3D RQE with good performance is more technically challenging. Although the similarity functions and the optimization algorithms used in 3D/3D registration can be used in 2D/3D registration with minimal modification, the failure pattern for 2D/3D registration is significant different. A 3D/3D registration is typically either successful or failed badly (i.e., far away from its optimal point in the transformation parameter space) because the objective function is smooth and converge quickly around the solution. The objective function for a rigid 2D/3D registration has much more local optima even in the close vicinity of the globally optimum solution. The use of a pattern classifier to identity those non-optimal solutions near the optimal solution is therefore much more challenging.

**II. METHODS**

**II.A Patient Data Acquisition**

In this study, we used the daily setup images of six randomly selected pediatric patients with ages ranging from 3 to 14. Patients selected for this study have previously consented to participation in our institutional IRB-approved protocol. All patients were diagnosed with brain tumors and have completed their proton therapy treatments in our institute. A pair of orthogonally projected kilovoltage (kV) x-ray images were acquired each day during the patient setup. The kV x-ray images, with 1152 x 1600 pixels, have pixel sizes of 0.18 x 0.18 mm^2^ and 0.21 x 0.21 mm^2^ for panel A and panel B, respectively. Treatment planning CT images were acquired with the pixel size of 0.75 x 0.75 mm^2^ in the axial plane and the slice thickness of 1 mm.

**II.B 2D/3D image registration**

The original x-ray images were first down-sampled at the ratio of 1:2 and then rigidly registered to their treatment planning CT images. Rigid registration between two x-ray images and the CT image were performed using an in-house GPU-based 2D/3D registration program. This program uses an iterative method to optimize the normalized mutual information (NMI) between the digitally reconstructed radiographs (DRRs) and the acquired x-ray images. The DRRs are computed in each iteration by ray-tracing a 3D CT image for a given set of transformation parameters using a graphics card. Each registration is based on the region-of-interest (ROI) defined on the x-ray images. The room coordinates of the x-ray images were determined by image overlays of physical cross-hair devices acquired simultaneously with the imaging subjects. The right-lateral and the posterior-anterior (PA) projection x-ray images of a selected patient are shown in Figs. 1a and 1b, respectively. Figures 1c and 1d show the calculated final DRRs when optimization was completed. Figures 1e-1h show the overlapped images of x-ray images and the edge images of DRRs. The edge images of DRRs were created by applying a Laplacian of Gaussian filter. Figures 1e and 1f show the images before registration and Fig. 1g and 1h the images after registration.

Each patient received 30 treatment fractions, thus producing a total of 30 unique registration tasks per patient. The rigid registrations computed three translational and three rotational parameters.

**II.C Training and Testing Data Preparation**

As described by Wu and Murphy[^12^](#_ENREF_12), a pattern classifier can be constructed and used to identify unsuccessful registration solutions that are caused by local minimum trapping or early termination. In this study, we applied this method to construct an RQE for 2D/3D registrations. Data preparation of the RQE involves: 1) sampling the parametrical space in the neighborhood of the point-of-solution, 2) computing a number of geometrical features from the sampled values, and 3) identifying the true category of a solution based on a user-defined error tolerance. We will describe these steps in details in the following sections.

*II.C.1 Parametrical Space sampling*

In the previous paper[^12^](#_ENREF_12), Wu and Murphy sampled the similarity measure values by varying one translational parameter at a time using two user-defined sampling step sizes, i.e., 1 mm and 5 mm. In this study, we sampled three rotational parameters in addition to the three translational parameters. This is because, for 2D/3D registrations, out-of-plane translations and rotations (with respect to the imaging planes) may change the similarity function value in different ways (e.g., different gradients) compared to the in-plane ones. The addition of rotational parameters will provide more information to the pattern classifier and potentially improve its performance. When we sampled the cost function, a normalized transform unit, the mean voxel displacement (MVD), was used for both translational parameters and rotational parameters in the parametrical space. Thus a unit change of a rotational or a translation parameter produces the same averaged voxel displacement within the ROI defined on the CT images. For the brain patients in this study, the ROIs on the CT images are rectangular cuboids that are approximately centered at the isocenter and include the whole brain. Those ROIs were manually defined by a user for each CT image. We investigated the impact of sampling step size on the performance of RQE by varying the step size from 1 mm to 30 mm with 5 mm increments.

*II.C.2 Geometrical Feature Calculations*

The distinctiveness of optimum (DO) and the mirror symmetry (MS) have been used by Wu and Murphy[^12^](#_ENREF_12) as geometrical features to evaluate their 3D/3D image registration solutions. The same geometrical features were used in this study but were computed separately for the translational samples and rotational samples.

The DO is based on our assumption that a global minimum generally has a deeper valley compared to that for a local minimum. The MS is based on our assumption that a global minimum has a relatively symmetrical profile centered at its minimum. The profile of a local optimum, on the other hand, is usually more irregular because of its limited capture range. Calculations of both features require the similarity function be sampled along the translational and rotational major axes in the parametrical space for a given step size. For each axis, the sampled points were centered at the current registration solution. The two geometrical features, as described by Wu and Murphy[^12^](#_ENREF_12), are briefly presented here.

i) Distinctiveness of optimum (DO)

As shown in Fig. 2, for a given sampling step size *s* in the transformation parameter space, the DO is given by

, (1)
where , is the average of the two sampled function values along the axis *i*. *N* is the number of the sampled axes. , , and are the similarity function values of the point , , and , respectively, on the axis *i*. Note that in our study the MVD is taken as the unit for the step size *s*.

ii) Mirror symmetry (MS)

As shown in Fig. 2, for a given step size *s*, the MS is defined as

, (2)
where all the symbols are defined the same as in Eq. (1). Note that the denominator in Eq. (2) may become zero under two trivial cases. One is for a flat local surface, in which case . The other is the case when , or . In this case, is not a global minimum. So in both cases, the solution can be easily classified as an unsuccessful registration.

As explained early, we computed DO and MS separately for translations and rotations, and denoted them as DO_trans_, DO_rot_, MS_trans_, and MS_rot_, respectively. These features were computed for each registration solution.

*II.C.3 Determination of True Categories*

We used the same method as proposed by Wu and Murphy[^12^](#_ENREF_12) to determine the true category of a registration solution. In this method, a “gold standard” registration solution was obtained by taking the best solution over 50 registration attempts. These 50 registration attempts differ by randomly selected initial transformation parameters that are centered at the initial setup position and within the range of ±5 mm and ±2° in the parametrical space. The best solution has the minimal final cost function (i.e., negated NMI). This best solution was visually verified using in-house visualization software.

Once the “gold standard” solutions were established, a new round of 20 registration repetitions was carried out for each registration problem to generate the training and test dataset for the RQE. These 20 registration repetitions also differ by randomly selected initial transformation parameters that are centered at the initial setup position and within the range of ±5 mm and ±2° in the parametrical space. This resulted in a total of 3600 registration solutions (i.e., 6 patients × 30 registration tasks per patient × 20 repetitions per registration task) to be used to train and test the RQE. By balancing the number of successful and unsuccessful registrations, the ranges of random perturbations to the initial transformation parameters were empirically chosen to be ±20 mm and ±8°.

The registration errors for these new solutions were quantified by calculating the MVD with respect to their “gold standard” solutions. As described by Skerl and colleagues,[^13^](#_ENREF_13) the MVD is equivalent to the Euclidean distance from the point of the current solution to the point of the “gold standard” solution in the normalized parametrical space. So the MVD of a registration solution can be calculated conveniently and compared to a user-defined error tolerance to determine the acceptance of this solution. In this paper, a MVD tolerance of 1 mm was used. We believe this choice is reasonable since one standard deviation (SD) of all successfully registration solutions is about 0.5 mm.

**II.D Training and Validation of the Neural Network**

The same two-layer feed-forward neural network as proposed by Wu and Murphy[^12^](#_ENREF_12) was used in this study. The network was implemented using the MATLAB Neural Network Toolbox (The MathWorks, Inc., Natick, MA). There are four inputs to the neural network. The inputs are the computed geometrical features—DO_trans_, DO_rot_, MS_trans_, and MS_rot_. There is only one single numerical output ranging from 0 to 1. An output greater than 0.5 is considered to belong to the category of unsuccessful registrations. Hyperbolic tangent sigmoid transfer functions were used in both the hidden layer and the output layer. To avoid the problem of over-fitting, effort has been taken to keep the network structure simple. We started with having 20 neurons in the hidden layer and gradually reducing the number of neurons. We found out the classifier still has very good performance when the number was reduced to two, but its performance deteriorated remarkably when there was only one neuron left in the hidden layer. Thus in our final neural network structure there are two neurons in the hidden layer. During the network construction, the scaled conjugate gradient optimization algorithm[^14^](#_ENREF_14) was used to minimize the mean sum-of-squares of the network output error. This method is based on the conjugate gradient method but was designed to avoid the time-consuming line search by combining with the model-trust region approach.

To overcome the problem of over-training and improve generality, the available data were divided into three subsets. Firstly, the data generated from each individual patient was chosen in turns as the test dataset. This is so called “leave-one-out” scheme. Then the rest of the data from five other patients were randomly divided into the training dataset (80%) and the validation dataset (20%). The training set was used for computing the gradient and updating the network weights and biases. After each training iteration the network was tested on the validation set. The test data had no effect on training and they provided an independent measure of network performance during and after training. Thus it provided us with a valid method to evaluate how the network was generalized to new patients. Two criteria, sensitivity and specificity, were used to evaluate the performance of the classifier.

**II.E Test of the Integrated Robust 2D/3D Registration System**

The RQE was integrated into our in-house GPU-based 2D/3D registration system. The same patient data set as mentioned above was used in this test. The RQE constructed with the first patient left out (arbitrarily chosen) were added to the 2D/3D registration system. Once a registration solution was reached, the RQE would determine whether the result was acceptable. If a solution was rejected, the registration method would restart with another trial but using a different set of randomly generated initial transform parameters. In this paper, we set the maximal number of trials as five. If the RQE failure to accept a solution after the maximal number of trials has been reached, we consider the registration results were not reliable and a manual registration was required. Here we intent to estimate the efficiency of this integrated system rather than evaluate the accuracy and performance of the system. Consider two scenarios. 1) If a registration solution was accepted after too many registration trials, then the system would have little efficiency. 2) If all solutions were accepted on their first trial, then we would not need an RQE. It should be noted that for the first case it may not means the RQE has bad performance. It may just mean the registration method failed to find a good solution. The method described in Section II.D is a better way to evaluate the performance of the RQE itself.

**III. RESULTS**

Figures 3a and 3b show the profiles of the cost function, i.e., negative NMI, along shifts in x, y, and z axes and rotations about x, y, and z axes from the point-of-solutions, i.e., the origins, in the parametrical space. The units of shifts and rotational deviations have been normalized to MVD. The profiles in Fig. 3a are centered on a typical good solution, where the curves on the positive side and the negative side are proximately symmetric. The profiles in Fig. 3b are centered on a typical bad solution, where some curves are not symmetric and the valleys for all curves are shallower than the corresponding ones in Fig. 3a. This demonstrated why DO and MS can be used to describe those two features.

The scatter plots of all data used in training, validation, and testing with 5 mm sampling size are shown in Figs. 4a and 4b. In Fig. 4a, the distinctiveness of optimum for translations (DOtrans) was plotted against the distinctiveness of optimum for rotations (DOrot). In Fig. 4b, the mirror symmetry for translations (MStrans) was plotted against the mirror symmetry for rotations (MSrot). The successful solutions are shown as blue circles and the unsuccessful solutions are shown as red crosses. As illustrated in 2D plots, there is not a clear boundary that can separate those two categories. It is reasonable to imagine that a simple boundary is not likely to be found in the 4D feature space that includes all four features.

To investigate the effect of sampling step size on the performance of the RQE, the RQEs were constructed and tested separately for various step sizes ranging from 1 mm to 30 mm with 5 mm increment. As described in the previous section, each time data from one of the six patients were chosen as the test data. The RQE was constructed using data from the rest of the patients for each given step size. Performance of the RQE evaluated using the test data from each chosen patient was described by sensitivity and specificity (as shown in Table 1). In Table 1, each column of entries is associated with a particular patient whose registration results were chosen as the test data. Based on our results, the RQE has the best overall sensitivity and specificity with a sampling step size of 5 mm.

The registration results of the integrated 2D/3D registration system are shown in Table 2. The trial-to-acceptance ratio (TAR), which is defined as the ratio between the total number of trails to the number of acceptance, for all patients, is equal to 1.48. This indicates the integrated system has about 48% overhead compared to the original system. The final acceptance ratio (FAR), which is defined as the number of accepted solution to the number of registration attempts (i.e., number of acceptances + number of final rejections), is 92.4%.

**IV. DISCUSSION**

Due to the similarity between the 2D/3D registration and the 3D/3D registration, this paper used the same training data generation, RQE generation and performance evaluation method as previously reported.[^12^](#_ENREF_12) However, we added features for rotational deviations in our 2D/3D RQE instead of utilizing only translational features. This is because, presumably, object translations and rotations about different axes may change the cost function at difference rates. This may not be a problem for the 3D/3D RQE because the cost function was calculated from 3D voxels. But the cost function for 2D/3D RQE is based on 2D pixels on two orthogonally projected images. Depending on their axes, a translation or a rotation can be in-plane or out-of-plane with respect to their projection image planes, which would result in different gradients in their cost function profiles. Generally speaking, including more non-redundant features in the classifier would potentially improve its performance with the cost of increased computation complexity, which is not significant in our case.

The performance of the classifier (e.g., sensitivity and specificity) depends on the threshold value that is used to categorize the neural network output. The optimal value for this threshold could be determined if the costs of making both the false positive and negative errors and the frequencies of making those errors were known.[^11^](#_ENREF_11) The choices of those parameters are application and user dependent and beyond the scope of this paper. In this study, a threshold value of 0.5 was used, which is based on the arbitrary assumption that the costs of making both errors are equal.

The optimal sampling size for the RQE in our study has been identified to be 5 mm. It should be noted that this value depends on many factors that could change the geometrical properties of the similarity function. Those factors may include treatment site, ROI definition, choice of similarity measure, DRR calculation method, and the imaging parameters such as pixel/voxel spacing, contrast, number of gray-levels, imaging artifacts and the noise level. However, for a specific clinical application, most of those factors are fixed. If we assume a relatively stable imaging system and have a protocol of ROI definition for creating the ROIs of the approximately same size and anatomical location among different patients, the optimal sampling size may be obtained per treatment site.

In this study, the NMI alone was used as the similarity measures and its geometrical properties are used by the classifier. It would be interesting to see if using a different similarity measure or using multiple measures simultaneously would improve the performance of the RQE.

The TAR (i.e., 1.48) we obtained indicates the RQE did effectively reject unsuccessful registration solutions. It also shows the integrated system requires about 48% more computation time compared to the original system. As mention before, this is not a major concern with the advance of the graphics card computation techniques. Of all the registration attempts, there are 92.4% cases that the solutions were finally accepted by the RQE before the maximal number of trials reached. Of all the rejected solutions, 90.9% are originated from registering images for patient 2. This is most likely due to the difference between the definitions of the ROI. A typical lateral projection x-ray image of patient 2 is shown in Figure 5a and its corresponding one for other patients shown in Figure 5b. A small portion of the posterior part of the cranial image was clipped out of the ROI for patient 2 because of the selection of the isocenter for this particular patient and the limited field-of-view (FOV) of the imaging system. For a scattering beam proton therapy system, the FOV of the x-ray imaging system corresponds to the choice of the treatment head (i.e., the snout size). Pediatric patients with brain tumor are typically treated with 25 cm snout. However, patient 2 was treated with 30x40 cm snout, which is very rare in our clinic. This results in a reduced FOV in the anterior-posterior direction in the lateral view. If this particular case is excluded from our dataset, the TAR will be reduced to 1.11 and FAR increased to 99.2%. With such a low TAR and high FAR, the RQE would greatly increase the robustness of the integrated 2D/3D registration system but at the same time without sacrificing much of the efficiency of the system.

Our results are based on very limited patient data and the cranial site only. More accurate statistical evaluation would require the test on more patient data. The applications on other treatment sites that have non-rigid deformations with larger magnitude would be more challenging but also interesting.

It would also be interesting to adopt a scheme to adaptively update the RQE using the registrations results when it is used clinically.[^15^](#_ENREF_15), [^16^](#_ENREF_16) The output of the RQE can be checked either online or offline by users. Erroneous decisions by the RQE can be corrected and fed back to the classifier to improve its future performance.

Two other more challenging but interesting extensions of this work are the applications of the RQE on multi-modality and/or deformable 2D/3D image registrations.[^17-20^](#_ENREF_17) The key issue is to find features that are good indicators of a successful registration. For example, a simple intensity-based similarity measure is not adequate for a deformable registration because a registration solution with high similarity measure value does not necessarily indicate the deformation is physically meaningful. The features must also be robust so that they do not vary greatly among different patients.

**VI. CONCLUSIONS**

Our patient study has demonstrated the proposed neural network based RQE had fairly good performance when used with the NMI in identifying unsuccessfully 2D/3D registrations for daily patient setup. This technique can be implemented clinically to improve the accuracy and the robustness of the patient setup system that relies on automated rigid 2D/3D registrations.

**References**

^1^ Bednarz G, Machtay M, Werner-Wasik M, Downes B, Bogner J, Hyslop T, Galvin J, Evans J, Curran W, Jr., Andrews D. Report on a randomized trial comparing two forms of immobilization of the head for fractionated stereotactic radiotherapy. Med Phys. 2009;36(1):12-7.

^2^ Cho B, Suh Y, Dieterich S, Keall PJ. A monoscopic method for real-time tumour tracking using combined occasional x-ray imaging and continuous respiratory monitoring. Phys Med Biol. 2008;53(11):2837-55.

^3^ Yan H, Cervino L, Jia X, Jiang SB. A comprehensive study on the relationship between the image quality and imaging dose in low-dose cone beam CT. Phys Med Biol. 2013;57(7):2063-80.

^4^ Kim J, Fessler JA, Lam KL, Balter JM, Ten Haken RK. A feasibility study of mutual information based setup error estimation for radiotherapy. Med Phys. 2001;28(12):2507-17.

^5^ Clippe S, Sarrut D, Malet C, Miguet S, Ginestet C, Carrie C. Patient setup error measurement using 3D intensity-based image registration techniques. Int J Radiat Oncol Biol Phys. 2003;56(1):259-65.

^6^ Rohlfing T, Russakoff DB, Denzler J, Mori K, Maurer CR, Jr. Progressive attenuation fields: fast 2D/3D image registration without precomputation. Med Phys. 2005;32(9):2870-80.

^7^ Ino F, Gomita J, Kawasaki Y, Hagihara K, presented at the The Fourth International Symposium on Parallel and Distributed Processing and Applications (ISPA'2006), Sorrento, Italy, 2006 (Springer-Verlag, Berlin, Germany).

^8^ Steininger P, Neuner M, Weichenberger H, Sharp GC, Winey B, Kametriser G, Sedlmayer F, Deutschmann H. Auto-masked 2D/3D image registration and its validation with clinical cone-beam computed tomography. Phys Med Biol. 2012;57(13):4277-92.

^9^ Spoerk J, Gendrin C, Weber C, Figl M, Pawiro SA, Furtado H, Fabri D, Bloch C, Bergmann H, Groller E, Birkfellner W. High-performance GPU-based rendering for real-time, rigid 2D/3D-image registration and motion prediction in radiation oncology. Z Med Phys. 2012;22(1):13-20.

^10^ Russakoff DB, Rohlfing T, Mori K, Rueckert D, Ho A, Adler JR, Jr., Maurer CR, Jr. Fast generation of digitally reconstructed radiographs using attenuation fields with application to 2D/3D image registration. IEEE Trans Med Imaging. 2005;24(11):1441-54.

^11^ Wu J, Samant SS. Novel image registration quality evaluator (RQE) with an implementation for automated patient positioning in cranial radiation therapy. Med Phys. 2007;34(6):2099-112.

^12^ Wu J, Murphy MJ. A neural network based 3D/3D image registration quality evaluator for the head-and-neck patient setup in the absence of a ground truth. Med Phys. 2010;37(11):5756-64.

^13^ Skerl D, Likar B, Pernus F. A protocol for evaluation of similarity measures for rigid registration. IEEE Trans Med Imaging. 2006;25(6):779-91.

^14^ Moller MF. A Scaled Conjugate-Gradient Algorithm for Fast Supervised Learning. Neural Networks. 1993;6(4):525-33.

^15^ Yan H, Yin FF, Zhu GP, Ajlouni M, Kim JH. Adaptive prediction of internal target motion using external marker motion: a technical study. Phys Med Biol. 2006;51(1):31-44.

^16^ Murphy MJ, Pokhrel D. Optimization of an adaptive neural network to predict breathing. Med Phys. 2009;36(40):40-7.

^17^ Rueckert D, Sonoda LI, Hayes C, Hill DLG, Leach MO, Hawkes DJ. Nonrigid registration using free-form deformations: application to breast MR images. IEEE Trans Med Imaging. 1999;18(8):712-21.

^18^ Hopp T, Dietzel M, Baltzer PA, Kreisel P, Kaiser WA, Gemmeke H, Ruiter NV. Automatic multimodal 2D/3D breast image registration using biomechanical FEM models and intensity-based optimization. Med Image Anal. 2013;17:209-18.

^19^ Naini AS, Patel RV, Samani A. CT-enhanced ultrasound image of a totally deflated lung for image-guided minimally invasive tumor ablative procedures. IEEE Trans Biomed Eng. 2010;57(10):2627-30.

^20^ Naini AS, Pierce G, Lee T, Patel RV, Samani A. CT image construction of a totally deflated lung using deformable model extrapolation. Med Phys. 2011;38:872-83.

**Figures and Figure Legends**


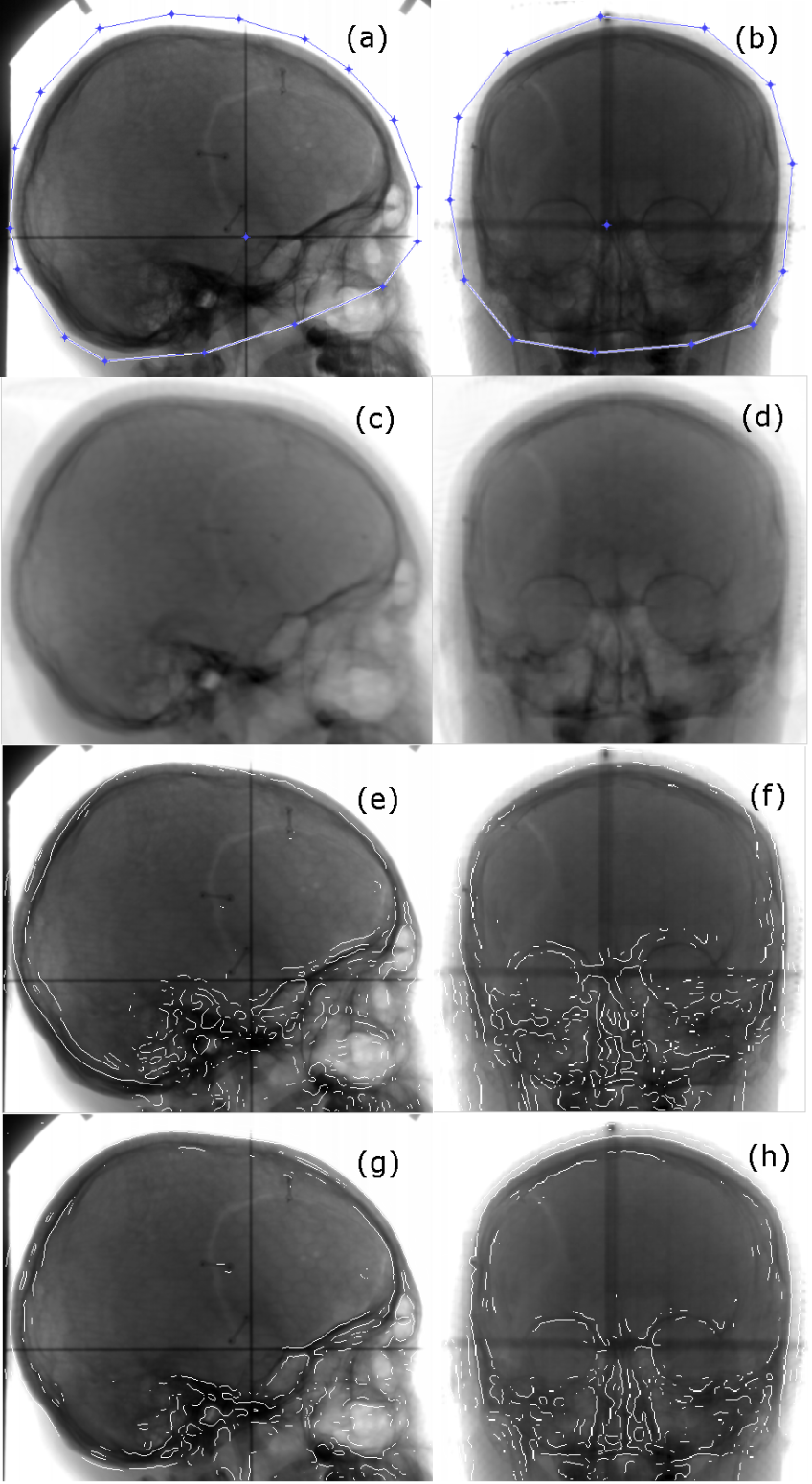


FIG. 1. The right-lateral view (first column) and the posterior-anterior view (second column) images of a selected pediatric patient. The setup verification x-ray images acquired just before treatment are shown in (a) and (b). The user-defined region-of-interests are indicated by the blue line segments. The calculated final DRRs when optimization was completed are shown in (c) and (d). X-ray images and the edge images of DRRs are overlapped before the registration [(e) and (f)] and after the registration [(g) and (h)] to show the effects of image registration.


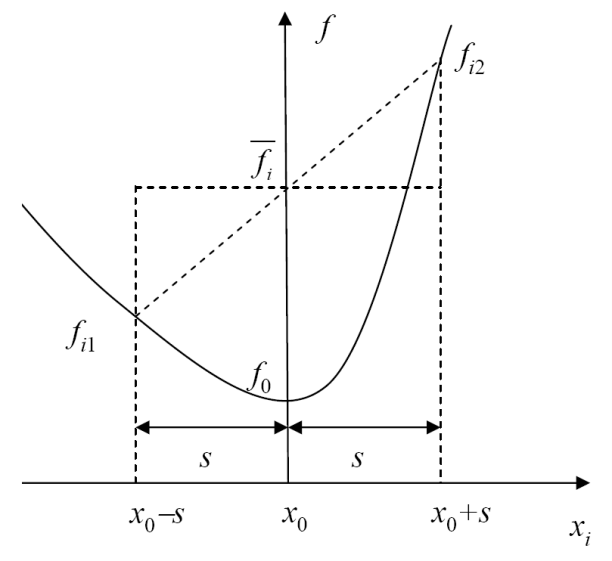


FIG. 2. Illustration of the definitions of the distinctiveness of optimum (DO) and the mirror symmetry (MS) (Ref. 12).


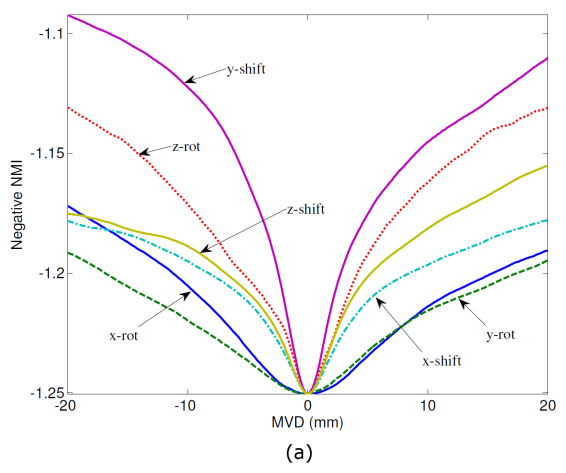

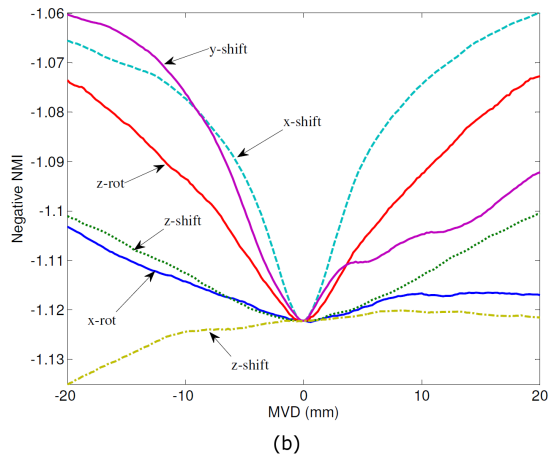


FIG. 3. Profiles of the cost function, i.e., negative NMI, along shifts in x, y, and z axes and rotations about x, y, and z axes from the point-of-solutions, i.e., the origins. The units of shifts and rotational deviations have been normalized to MVD. The profiles of a typical good solution and a bad solution are shown in (a) and (b), respectively.


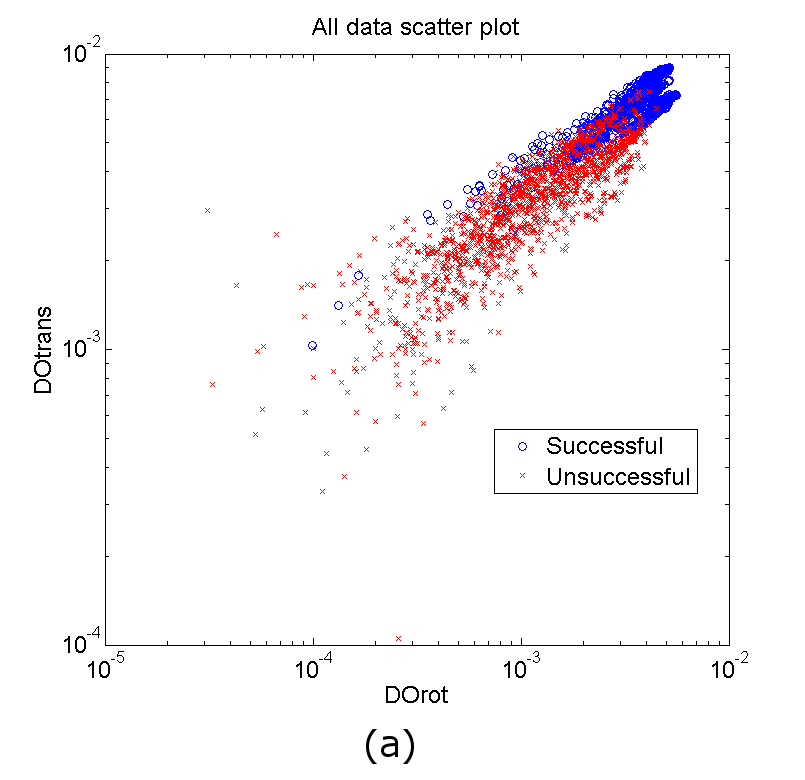

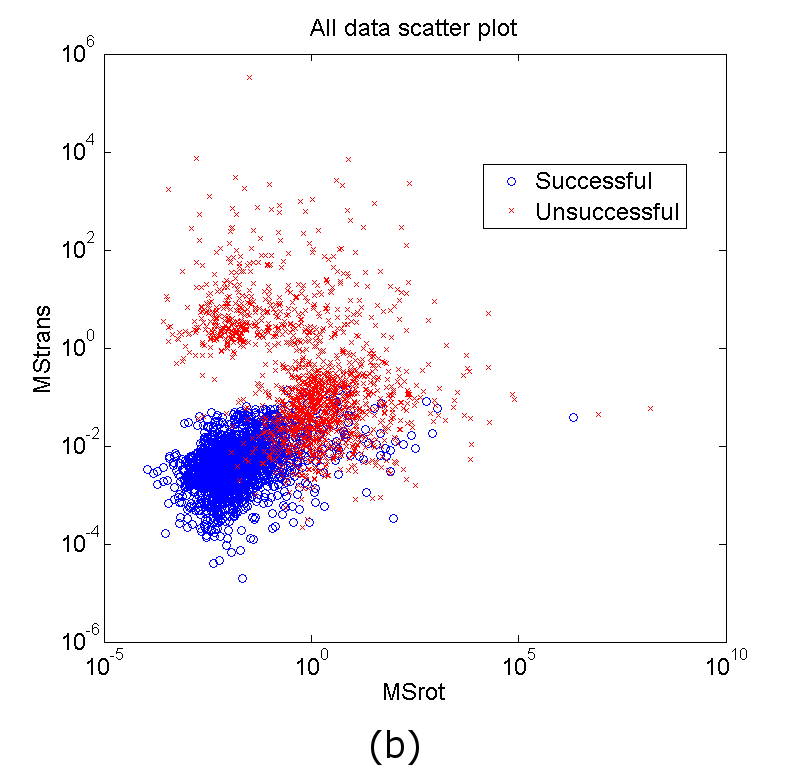


FIG. 4. Scatter plots of (a) DOtrans v.s. DOrot and (b) MStrans v.s. MSrot of all data with 5 mm sampling size. The successful solutions are shown as blue circles and the unsuccessful solutions as red crosses.


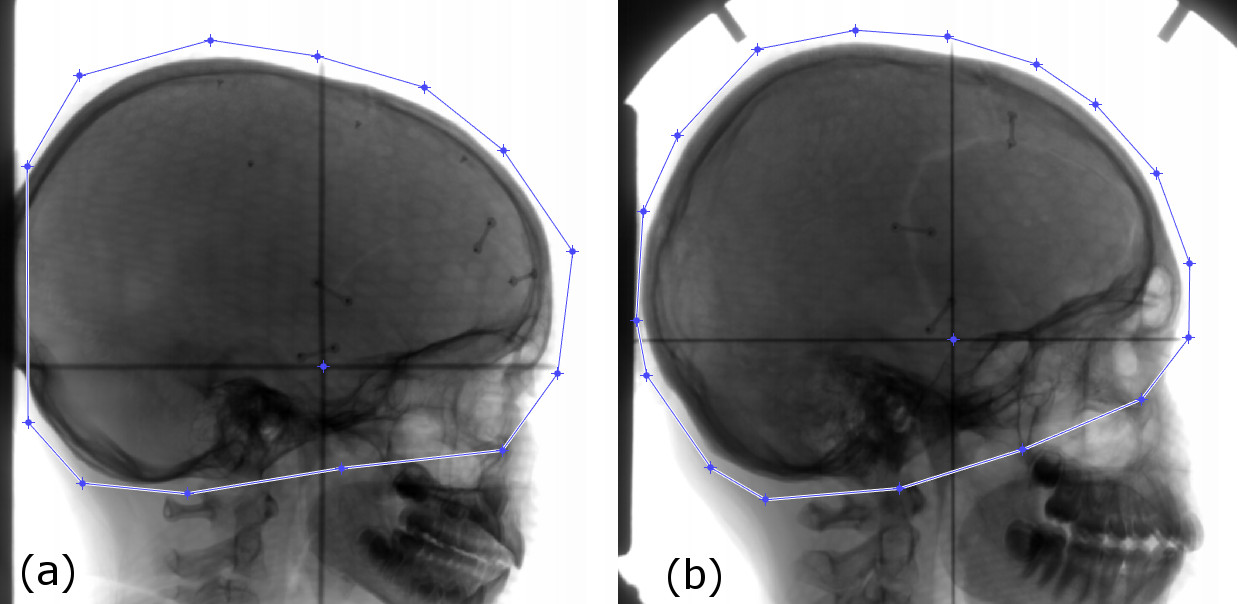


Figure 5. A typical right-lateral projection x-ray image of (a) patient 2 and (b) a patient other than patient 2.

**Tables and Captions**

Table 1. Sensitivities and specificities of the classifier for various sampling step sizes and test datasets.

| Sampling step size (mm) |  | Test dataset | | | | | |
| --- | --- | --- | --- | --- | --- | --- | --- |
|  |  | Patient 1 | Patient 2 | Patient 3 | Patient 4 | Patient 5 | Patient 6 |
| 1 | Sensitivity | 0.894 | 0.957 | 0.889 | 0.840 | 0.929 | 0.680 |
|  | Specificity | 0.919 | 0.764 | 0.970 | 0.987 | 0.946 | 0.953 |
| 5 | Sensitivity | 0.940 | 0.964 | 0.917 | 0.905 | 0.915 | 0.865 |
|  | Specificity | 0.933 | 0.797 | 0.976 | 0.990 | 0.980 | 0.927 |
| 10 | Sensitivity | 0.95 | 0.939 | 0.877 | 0.945 | 0.868 | 0.892 |
|  | Specificity | 0.923 | 0.721 | 0.988 | 0.984 | 0.98 | 0.927 |
| 15 | Sensitivity | 0.929 | 0.946 | 0.873 | 0.876 | 0.872 | 0.865 |
|  | Specificity | 0.926 | 0.658 | 0.979 | 0.990 | 0.980 | 0.927 |
| 20 | Sensitivity | 0.950 | 0.964 | 0.897 | 0.891 | 0.875 | 0.865 |
|  | Specificity | 0.916 | 0.605 | 0.979 | 0.990 | 0.980 | 0.925 |
| 25 | Sensitivity | 0.957 | 0.950 | 0.877 | 0.887 | 0.872 | 0.883 |
|  | Specificity | 0.899 | 0.625 | 0.979 | 0.990 | 0.980 | 0.930 |
| 30 | Sensitivity | 0.950 | 0.928 | 0.829 | 0.880 | 0.879 | 0.901 |
|  | Specificity | 0.916 | 0.658 | 0.982 | 0.993 | 0.980 | 0.927 |

Table 2. Registration results for the integrated robust 2D/3D registration system.

|  | Patient 1 | Patient 2 | Patient 3 | Patient 4 | Patient 5 | Patient 6 | Total |
| --- | --- | --- | --- | --- | --- | --- | --- |
| # of Acceptance | 317 | 190 | 320 | 310 | 320 | 290 | 1747 |
| # of Trials | 359 | 850 | 334 | 388 | 349 | 298 | 2578 |
| # of Final Rejections | 3 | 130 | 0 | 10 | 0 | 0 | 143 |
